# Proteomics computational analyses suggest that baculovirus GP64 superfamily proteins are class III penetrenes

**DOI:** 10.1186/1743-422X-5-28

**Published:** 2008-02-18

**Authors:** Courtney E Garry, Robert F Garry

**Affiliations:** 1 Department of Biology, The University of Texas at Austin, Austin, Texas, 78701, USA; 2 Department of Microbiology and Immunology, Tulane University Heath Sciences Center, New Orleans, Louisiana, 70112, USA

## Abstract

**Background:**

Members of the *Baculoviridae *encode two types of proteins that mediate virus:cell membrane fusion and penetration into the host cell. Alignments of primary amino acid sequences indicate that baculovirus fusion proteins of group I nucleopolyhedroviruses (NPV) form the GP64 superfamily. The structure of these viral penetrenes has not been determined. The GP64 superfamily includes the glycoprotein (GP) encoded by members of the *Thogotovirus *genus of the *Orthomyxoviridae*. The entry proteins of other baculoviruses, group II NPV and granuloviruses, are class I penetrenes.

**Results:**

Class III penetrenes encoded by members of the *Rhabdoviridae *and *Herpesviridae *have an internal fusion domain comprised of beta sheets, other beta sheet domains, an extended alpha helical domain, a membrane proximal stem domain and a carboxyl terminal anchor. Similar sequences and structural/functional motifs that characterize class III penetrenes are located collinearly in GP64 of group I baculoviruses and related glycoproteins encoded by thogotoviruses. Structural models based on a prototypic class III penetrene, vesicular stomatitis virus glycoprotein (VSV G), were established for Thogoto virus (THOV) GP and *Autographa california *multiple NPV (AcMNPV) GP64 demonstrating feasible cysteine linkages. Glycosylation sites in THOV GP and AcMNPV GP64 appear in similar model locations to the two glycosylation sites of VSV G.

**Conclusion:**

These results suggest that proteins in the GP64 superfamily are class III penetrenes.

## Introduction

The entry of enveloped animal viruses into target cells occurs via fusion of the viral membrane with a cellular membrane. Penetrenes are viral membrane proteins that mediate penetration into the host cell. The penetrenes of enveloped animal viruses can be divided on the basis of common structural motifs into at least three classes. Orthomyxoviruses, retroviruses, paramyxoviruses, arenaviruses, and coronaviruses encode class I penetrenes [1-6], which are also known as class I viral fusion proteins or α-penetrenes. Class I penetrenes contain a "fusion peptide," a cluster of hydrophobic and aromatic amino acids located at or near the amino terminus, an amino terminal helix (N-helix, HR1), a carboxyl terminal helix (C-helix, HR2), usually an aromatic amino acid (aa) rich pre-membrane domain and a carboxyl terminal anchor [1,7,2,9]. Envelope glycoprotein (E) and envelope glycoprotein E1 encoded respectively by members of the *Flavivirus *genus of the *Flaviviridae *and the *Alphavirus *genus of the *Togaviridae *are class II penetrenes (β-penetrenes) [10-12]. Class II penetrenes possess three domains (I-III) comprised mostly of antiparallel β sheets, a membrane proximal α-helical stem domain and a carboxyl terminal anchor. The fusion loops of class II penetrenes are internal and located in domain II. Members of the two other *Flaviviridae *genuses, *Hepaciviruses *and *Pestiviruses*, appear on the basis of proteomics computational analyses to encode truncated class II penetrenes [13]. Proteomics computational analyses suggest that the carboxyl terminal glycoproteins (Gc) of bunyaviruses, and similar proteins of tenuiviruses and a group of *Caenorhabditis elegans *retroviruses, are also class II penetrenes [14]. Additional evidence that bunyavirus Gc are class II penetrenes has been provided [15,16].

Recent studies have provided evidence for a third class of viral penetrenes (class III or γ-penetrenes). The entry glycoprotein (G) of vesicular stomatitis virus (VSV), a rhabdovirus, contains a fusion domain comprised of β sheets, other β sheet domains, an extended α-helical domain, a membrane proximal α-helical stem domain and a carboxyl terminal anchor [17,18]. On the basis of sequence similarity it is likely that G of other members of the *Rhabdoviridae *are also class III penetrenes. Although larger, glycoprotein B (gB) of herpes simplex virus type 1 (HSV-1) and by sequence similarity gB of other herpesviruses, were unexpectedly demonstrated to share several structural features with VSV G [19]. The extended α-helices in the post-fusion forms of G and gB are involved in trimerization, as is well documented for α-helices in the post-fusion structures of class I penetrenes. The fusion domains of rhabdovirus G and herpesvirus gB are very similar structurally to the fusion domains of class II penetrenes [17-20]. Therefore, class III penetrenes may share a common progenitor(s) with members of other penetrene classes.

Members of the *Baculoviridae *are enveloped double-stranded DNA viruses of arthropods that are subdivided into two genuses, *Nucleopolyhedroviru*s (NPV) and *Granulovirus *(GV). NPV are further subdivided into group I and II. Baculoviruses encode two distinct penetrenes [21,22]. Entry proteins of group I NPV are all approximately 64 kilodalton glycoproteins (GP64), and are referred to collectively as GP64 superfamily proteins [23]. Group II NPV and GV encode entry proteins referred to as fusion proteins (F) [22,24]. Group I NPV often encode both GP64 and F homologues, although in these viruses F is nonfunctional. *Autographa california *multiple NPV (AcMNPV) lacking GP64 can be pseudotyped by the F protein of *Spodoptera exigua *MNPV [25], suggesting that F of group II NPVs and GV can serve as a functional analog of GP64. However, GP64 cannot serve as an analog of F [26]. Baculovirus F are class I penetrenes. Structural similarities exist between baculovirus F, the envelope glycoproteins of insect retroviruses (errantoviruses), the envelope glycoprotein of the gypsy retrotransposon of *Drosophila melanogaster *and other class I penetrenes [24]. Like other class I penetrenes, baculovirus F is present in virions as a homotrimer and synthesized as a precursor (F_0_), which is subsequently cleaved by furin-like proteases into subunits F_1 _and F_2 _[27,28]. Prior studies have not revealed structural relationships between baculovirus GP64 proteins and other penetrenes.

Thogoto virus (THOV) is a tick-transmitted virus, which is classified in the *Thogotovirus *genus of the *Orthomyxoviridae*. The genome of THOV comprises six segments of single-stranded, negative-sense RNA. The fourth largest RNA segment of THOV encodes a glycoprotein (GP) that has significant similarity with corresponding proteins of Dhori, Araguari, and Batken viruses and other thogotoviruses. Thogotovirus GP do not share significant sequence similarities with the class I penetrenes, hemagglutinin 2 (HA2) or hemagglutinin-esterase 2 (HE2), encoded by members of the three influenza virus genuses (types A, B and C) of the *Othomyxoviridae *or the fusion (F) protein or HE2 encoded by members of the *Isavirus *genus, the fifth orthomyxovirus genus [29]. However, thogotovirus GP share significant sequence similarity with baculovirus GP64, and are included in the GP64 superfamily [30,31]. Here, we present the results of proteomics computational analyses that suggest that GP64 superfamily members are class III penetrenes.

## Materials and Methods

### Sequences

Sequence and structural comparisons were performed for THOV strain SiAr 126 envelope glycoprotein precursor (THOV GP, accession number P28977), the AcMNPV GP64 superfamily protein (AcMNPV GP64, P17501) and other GP64 superfamily members. Representatives of G from six genera of the *Rhabdoviridae *were also used for sequence and structural comparisons: *Vesiculovirus*: VSV strain Indiana (AAA48370); *Lyssavirus*: rabiesvirus strain street (AAA47211); *Ephemerovirus*: bovine ephemeral fever virus structural G (P32595) and nonstructural G (P32596); *Novirhabdovirus*: infectious hematopoietic necrosis virus (CAA61498); *Cytorhabdovirus*: lettuce necrosis yellows virus glycoprotein (LYP425091); *Nucleorhabdovirus*: rice yellow stunt virus (AB011257) and an unclassified rhabdovirus: Taastrup virus (AY423355). We also compared GP64 superfamily members to penetrenes of representative members of the *Herpesviridae*, *Flaviviridae, Togaviridae*, and *Bunyaviridae*. Comparisons of F from ISAV strain RPC/NB 98-049-1 (ABE98322) and strain RPC/NB 98-0280-2 (ABE02810), F from *Spodoptera exigua *MNPV (AAF33539) and retrovirus-related Env polyprotein from transposon gypsy (P10403) were made to HA from influenza A virus strains A/WSN/1933 (H1N1, AAA3209), A/Aichi/2/1968 (H3N2, AAA43178), A/udorn/1972 (H3N2, ABD79032), A/guinea fowl/Italy/330/97 (H5N2, AF194991), A/chicken/Korea/S20/2004 (H9N2, AAV68031) and influenza B virus, strain B/Texas/37/1988 (ABN50602). Comparisons were also made amongst HE of influenza C virus strains Yamagata/9/88 (BAA06094) and C/Johannesburg/1/66 (CAL69520), ISAV strain T91/04 (AAY40756), human coronavirus OC43 strain ATCC VR-759 (AAR01014) and human torovirus (AAF00614).

### Proteomics computational methods

Methods developed by William Gallaher and coworkers to derive models of viral surface glycoproteins have been described previously [7,3,2,5]. William Pearson's LALIGN program, which implements a linear-space local similarity algorithm, was used to perform regional alignments. PHD (Columbia University Bioinformatics Center), which is part of the ProteinPredict suite was the preferred method of secondary structure prediction. Domains with significant propensity to form transmembrane helices were identified with TMpred (ExPASy, Swiss Institute of Bioinformatics). TMpred is based on a statistical analysis of TMbase, a database of naturally occurring transmembrane glycoproteins [32]. Sequences with propensity to interface with a lipid bilayer were identified with Membrane Protein e**X**plorer version 3.0 from the Stephen White laboratory using default settings [33], which can be used to calculate scores on the Wimley-White interfacial hydrophobicity scale (WWIHS) [34]. MacPymol [35] was used to render 3D models of VSV G (2cmz.pdb) and HSV-1 gB (2gum.pdb) in the post-fusion configurations. These models were extrapolated to THOV GP and AcMNPV GP64 using Photoshop (Adobe) and Freehand (Macromedia).

## Results

### Similar sequences and common structural/functional motifs are located collinearly in VSV G, THOV GP and AcMNPV GP64

Gallaher and co-workers employed the fusion peptide and other conserved features in combination with computer algorithms that predict secondary structure, to construct working structural models of several viral entry/fusion proteins, collectively referred to here as class I penetrenes [7,2,3,5,6]. This strategy has proven to be highly predictive of structures solved later by X-ray crystallography [4,36]. Gallaher's strategy, supplemented with increasingly robust proteomics computational tools, can also be applied to discovery of potential structures of viral penetrenes that belong to class II [13,14]. Here, we apply these methods to THOV GP and AcMNPV GP64, representative members of the GP64 superfamily.

**Figure 1:**
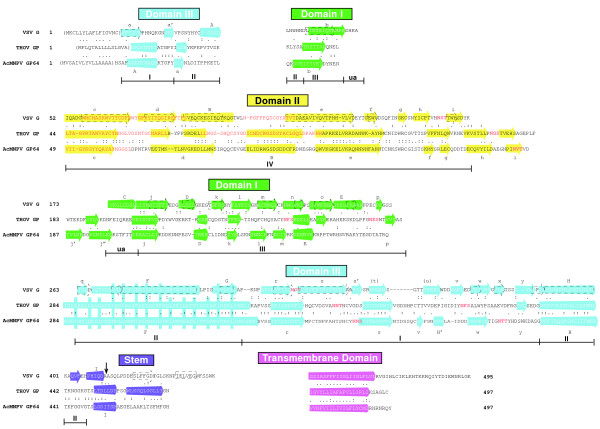
**Collinear arrangement of similarities in THOV GP, AcMNPV GP64 and VSV G.** A common domain nomenclature for class III penetrenes is utilized: domain I (green), domain II (yellow), domain III (blue), and stem domain (indigo). The domain numbering originally proposed is also indicated [17]. UA represents "hinge" aa not assigned to domains in VSV G in the prior scheme. Sequences with significant WWIHS scores in the fusion domain (II) were identified by MPeX and colored red. Hydrophobic transmembrane domains (violet) were predicted using TMpred. The post fusion secondary structure of VSV G as solved and numbered by Roche and coworkers [17] is depicted with α-helices as cylinders and β-sheets as arrows. The α-helices predicted by PHD In THOV GP and AcMNPV GP64 are indicated similarly. β-sheets (t) and (u) of VSV G are not present in the protein data base structure (2cmz.pdb). In VSV G, α-helices predicted by PHD are indicated by dashed boxes and predicted β-sheets are identified with dashed arrows. Amino acids are numbered beginning after the putative signal sequences enclosed in parentheses. In the alignments (:) refers to identical amino acids. (.) refers to chemically similar amino acids. Plum amino acids: N-glycosylation sites.

The PHD algorithm predicts protein secondary structure from multiple sequence alignments by a system of neural networks, and is rated at an expected average accuracy of 72% for three states, helix, strand and loop. Application of PHD to VSV G, a prototypic class III penetrene [17], reveals that predicted secondary structures (Fig. 1, α-helix and β-sheets depicted with dashed lines) closely correspond to the structures determined by X-ray crystallography (colored cones and arrows, accuracy; 77.5%). The PHD algorithm predicts that there is an extended α-helix in THOV GP (aa 284–338; blue cone) and AcMNPV GP64 (aa 284–340). With the exception of this extended α-helix, the ectodomains of the GP64 superfamily proteins are comprised mostly of β-sheets (colored arrows). Another domain readily identifiable with computational tools in THOV GP and AcMNPV GP64 is the carboxyl terminal transmembrane anchor. TMpred, an algorithm that identifies possible transmembrane helices, assigns significant scores (> 500 is statistically significant) to THOV GP aa 471–491 (score: 2428) and AcMNPV GP64 aa 470–490 (3030), which suggests that these sequences represent the transmembrane anchors (violet cones). PHD analyses also predict the presence of an α-helical stem domain with several aromatic aa (indigo cones) in THOV GP (aa 442–472) and AcMNPV GP64 (aa 441–471) prior to the transmembrane anchor, a feature present in both class II and III penetrenes [37-39,18].

**Figure 2:**
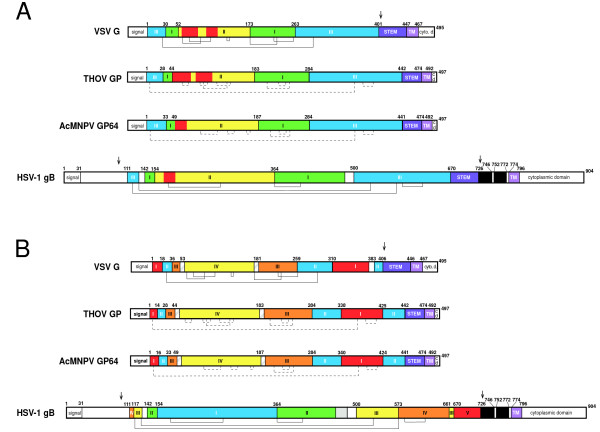
**Similar linear arrangement of putative domain structures of THOV GP and AcMNPV GP64 compared to domain structures of VSV G and HSV-1 gB.** Amino acids are numbered beginning after the putative signal sequences in VSV G, but at the beginning of the signal sequence of HSV-1 gB. Arrows indicate G and gB truncations of the forms used for crystallography. Solid lines represent cysteine bonding in VSV G and HSV-1 gB. Black boxes represent hydrophobic regions, with violet representing the transmembrane anchor (TM) [51]. Dashed lines represent potential cysteine bonding in THOV GP and AcMNPV GP64. Panel A: class III penetrene domain nomenclature and coloring as in Fig. 1. Panel B: domain nomenclature and color coding schemes used previously for VSV G [17] and for HSV-1 gB [19]. Hatched boxes in VSV G represent "hinge" aa not assigned to domains.

The structural determinations of VSV G and HSV-1 gB were performed by independent groups and although it was established that similar domains/structures are present, a consistent domain nomenclature for these class III penetrenes was not used (compare Fig. 2A with Fig. 2B) [17,19]. The fusion domains of class II and III penetrenes have a highly similar structure. Therefore, a class III domain nomenclature is used here that can apply to both rhabdovirus G and herpesvirus gB and assigns domain II (IV in the VSV G nomenclature of Roche et al. [17], I in the HSV-1 gB nomenclature of Heldwein et al. [19]) as the class III fusion domain as in class II penetrenes. In addition to minor adjustments in the ends of domains, the current class III penetrene numbering also combines two interacting domains into domain III (I + II in Roche's VSV G nomenclature, III + IV in Heldwein's HSV-1 gB nomenclature). The fusion domains of all class II or III penetrenes contain 1 or 2 prominent fusion loops, which give significant scores on the WWIHS [34]. Sequences with positive WWIHS have a high potential to interface with or disrupt lipid membranes, and therefore are key features of viral penetrenes. Another feature of the fusion domains of class II and III penetrenes is the presence of several dicysteine bonds, which appear to stabilize the overall domain architecture. Regions in THOV GP (aa 44–182) and AcMNPV GP64 (aa 49–186) with 6 or 8 cysteine residues, plus 1 or 2 sequences with positive WWIHS scores (Fig. 1, red letters) are likely to represent the fusion domains.

A prominent feature of class III penetrenes is an extended α-helix beginning near the carboxyl terminal third of the ectodomain (domain III), which is involved in trimerization of the post-fusion structure [17,19]. The extended α-helices predicted by PHD in THOV GP and AcMNPV GP64 correspond to this location. As noted previously [40], the sequence of the predicted helices is consistent with that of a leucine zipper (mostly leucines or isoleucines in the first and fourth positions of seven amino acid repeats), as is the case for both VSV G (Fig. 1, blue bars) and HSV-1 gB (not shown). The α-helices in the GP64 proteins are predicted to be several helical turns longer than the major helix (helix H) of the post-fusion structure of VSV G, but comparable in length to the major α-helix of HSV-1 gB.

Sequence similarities between VSV G, THOV GP and AcMNPV GP64 do not permit alignment by computational methods alone. However, using the regions of local structural similarity including the putative fusion domain/loops, extended α-helices and transmembrane domains, all of which are collinear, alignments between VSV G, THOV GP and BV GP64 are proposed (Figs. 1, 2). These alignments support assignment of a common domain architecture for these proteins. The proposed domains of these GP64 superfamily members are also collinear with analogous domains of herpesvirus gB, the other prototypic class III penetrene (Fig. 2).

### Structural models of THOV GP and AcMNPV GP64

**Figure 3:**
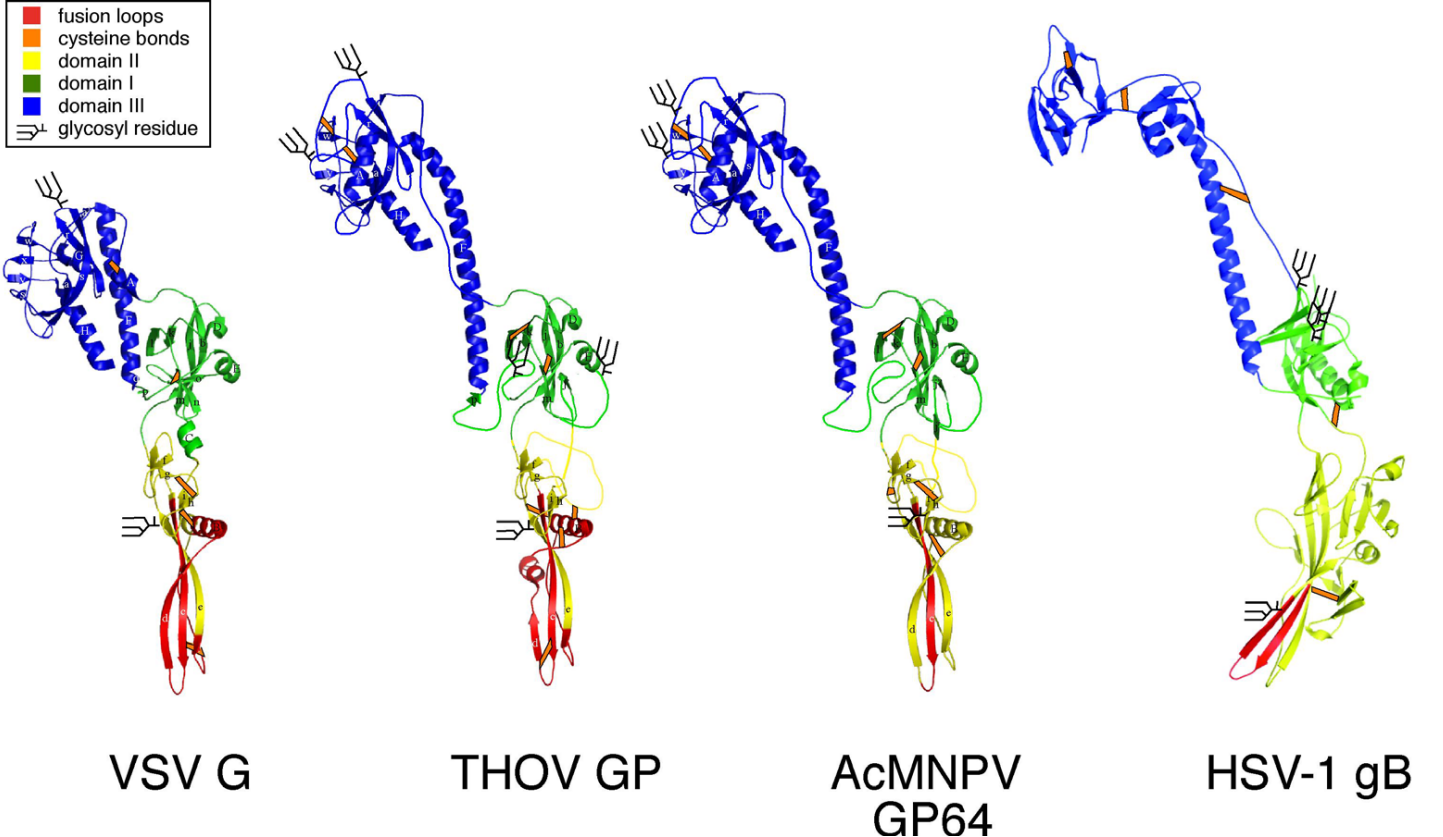
**Models of THOV GP and AcMNPV GP64 based on the X-ray crystallographic structure of VSV G.** The predicted structures of THOV GP and AcMNPV GP64 were fit to the post-fusion structure of VSV G [17]. Secondary structures for THOV GP and AcMNPV GP64 were predicted by PHD or by alignment to VSV G. The structure of HSV-1 gB [19] is shown for comparison. Domain coloring as in Fig. 1 and Fig. 2A. Orange/black lines: dicysteine linkages as in Fig. 2. Black stick figures: N-glycosylation sites.

Cysteine residues are usually the most conserved aa within a protein family because disulfide bonds between cysteines are critical determinants of secondary structure. The cysteines of class III (and class II) penetrenes determined by X-ray crystallography are arranged such that most disulfide bonds are formed between cysteine residues within the same domain (Fig 2). To determine the plausibility of the proposed alignment, models of THOV GP and AcMNPV GP64 scaffolded on the structure of VSV G in the post-fusion (low pH) configuration [17] were constructed (Fig. 3). The alignments between VSV G, THOV GP and AcMNPV suggest that these penetrenes may have a similar structure. Therefore, putative structures in the GP64 superfamily members are depicted as in VSV G. The proposed THOV GP and AcMNPV GP64 models are based principally on the structural predictions of PHD, the most robust secondary structure prediction algorithm used. These results provide evidence that the 6 or 8 cysteines in the portion of THOV GP and AcMNPV GP64 that align with the VSV G fusion domain (domain II) potentially bond with each other. Such linkages can stabilize the fusion loops as occurs in both class II and III penetrenes. There are also plausible intradomain linkages that can form between each of the other cysteines in THOV GP and AcMNPV GP64.

The results of these analyses suggest that the locations of the glycosyl residues may be conserved in class III penetrenes. Domain I of VSV contains a consensus glycosylation motif (NXS/T) between β-sheets h and I (Fig. 1). The other glycosylation site in VSV G is located between β-sheets r and s in domain III. THOV GP, AcMNPV and other GP64 superfamily members have similarly located glycosylation sites on or between predicted β-sheets corresponding to VSV G β-sheets h and i and r and s (Figs. 1, 3).

The THOV GP and AcMNPV GP64 structural models are not intended as definitive structural predictions. Rather, there are many possible alternatives to the secondary and tertiary structures and the cysteine linkages of these and other GP64 superfamily members. The modeling does establish that feasible structures exist that are consistent with the secondary structure predictions and with the assignment of GP64 superfamily members as class III penetrenes. The results of this structural modeling also provide further support for the proposed alignments of VSV G with THOV GP and AcMNPV GP64.

### Alignment of isavirus F with influenza A and virus hemagglutinin and influenza C virus hemagglutinin-esterase

**Figure 4:**
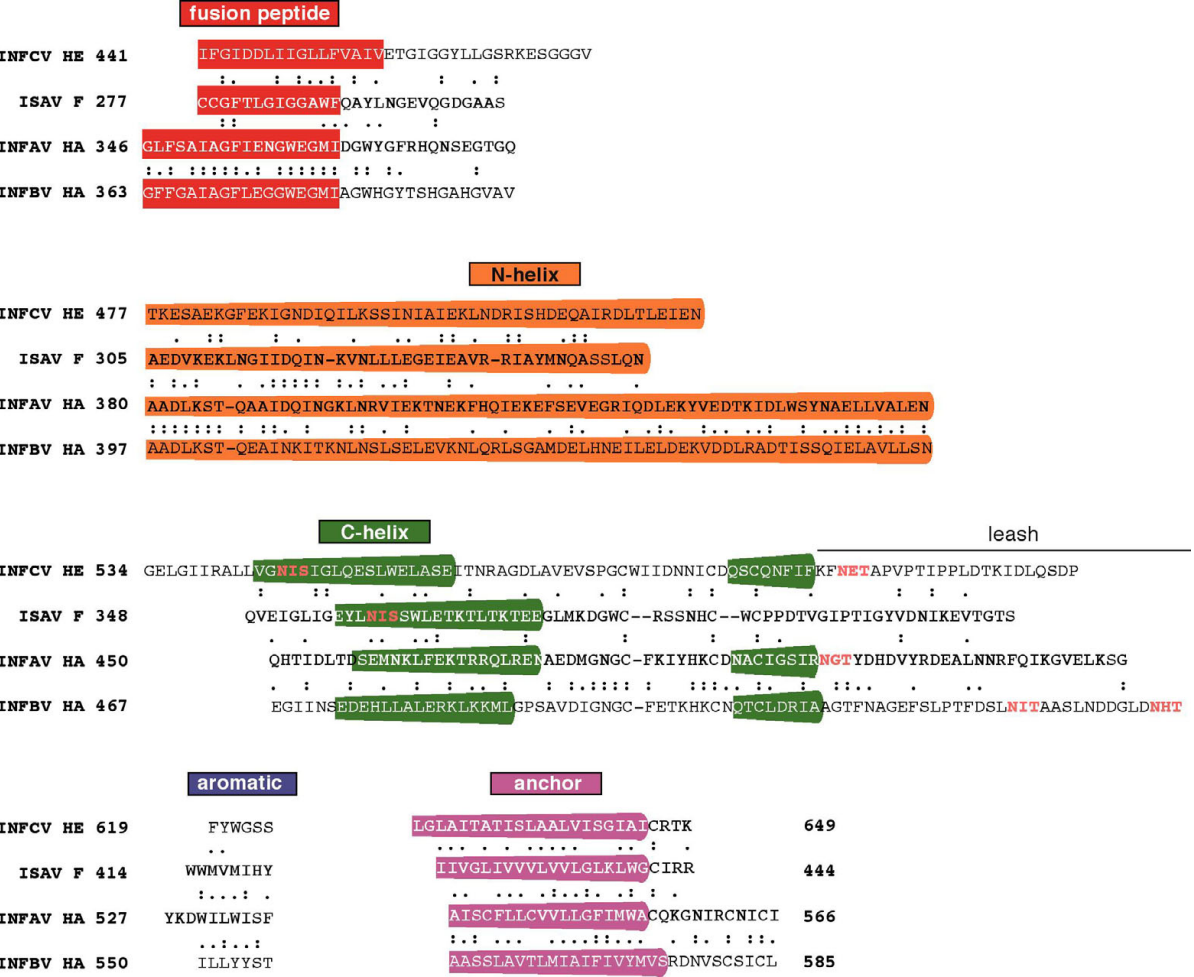
**Alignment of the class I penetrenes of orthomyxoviruses.** Alignment of HE2 of influenza C virus with HA2 of influenza A and B virus and F_1 _of ISAV. Fusion peptide red, amino terminal helix (N-helix) orange, c-terminal helix (green), aromatic domain (indigo). Hydrophobic transmembrane domains (violet).

Our computational analyses and molecular modeling studies suggest that thogotovirus penetrenes are structurally distinct from penetrenes encoded by viruses in the three other genuses of the *Orthomyxoviridae*, influenza A, B and C viruses. The fifth genus in the *Orthomyoviridae*, *Isavirus*, is represented by infectious salmon anemia virus (ISAV). ISAV encodes two glycoproteins, one of which (HE) has hemagglutinin and esterase activities [41]. The other ISAV glycoprotein is a class I penetrene designated the fusion protein (F). Previous studies by Aspehaug and coworkers indicated that like other class I penetrenes, ISAV F_1 _is produced by cleavage of a precursor (F_0_) that exposes a hydrophobic fusion peptide near the amino terminus [29]. To further investigate the relationship of isavirus F_1 _to the class I penetrenes of other orthomyxoviruses sequence comparisons and molecular modeling were conducted. While the alignment we report here is somewhat different than that proposed previously [29], it is also consistent with the designation of ISAV F_1 _as a class I penetrene (Fig. 4). ISAV F_1 _is shorter in overall length than HA2 of either influenza A or B viruses or HE2 of influenza C viruses, but comparable in length to certain class I penetrenes, such as glycoprotein 2 of Ebola virus. PHD reveals the presence of an extended α-helical domain that corresponds to the N-helix of influenza A and B viruses. The N-helix of influenza A and B virus HA2 features a leucine zipper, which is involved in trimerization. Other similarities include the presence of a conserved cluster of three cysteine residues (ISAV F aa 382–390) and an aromatic pre-anchor domain (aa 414–421). Of note is the location of the C-helix in ISAV F_1_. In comparison to most other class I penetrenes, the C-helices of influenza viruses in HE2 or HA2 are shorter and located more distally from the C-terminus. A sequence termed the leash is located between the C-helix and the aromatic domain. The formation of the post-fusion configuration of influenza A, B and C virus HA2 or HE2 is best described by a leash-in-grove mechanism, rather than by a six-helix bundle mechanism as in most other class I penetrenes [42]. The location of the C-helix in ISAV F_1 _suggests that this penetrene shares a common progenitor with HA or HE of influenza A, B and C viruses, and mediates fusion/penetration by a leash-in-grove mechanism.

## Discussion

Proteomics computational analyses suggest that GP64 superfamily proteins are class III penetrenes. Each of the major features common to class III fusion proteins are present in THOV GP and AcMNPV GP64, including internal fusion loops, an extended α-helical domain, a stem domain and a carboxyl terminal transmembrane domain. These features are located collinearly with these features in VSV G, a prototypic class III penetrene [17,18]. On the basis of sequence similarities among the GP64 superfamily members it is likely that all are class III penetrenes. Previous studies have suggested a role for the putative extended α-helix and the leucine zipper motif in GP64 mediated fusion/entry, but did not assign GP64 to any penetrene class [40,43]. Our results do not corroborate the previous conclusion [43] that a 6 aa sequence (AcMNPV aa 209–214 in Fig. 1) may be the GP64 fusion peptide. Structural models, which include feasible cysteine linkage maps, could be established for THOV GP and AcMNPV GP64. The fusion domains of THOV GP and AcMNPV GP64 appear to be stabilized by cysteine bonds and to contain one or more loops with positive WWIHS scores, features that are characteristic of the fusion domains of both class II and III penetrenes. Glycosylation sites in THOV GP and AcMNPV GP64 appear in similar model locations to the two glycosylation sites of VSV G. Whether or not the secondary and tertiary folding of GP64 superfamily members conform to the domain structure of class III penetrenes will require x-ray crystallographic or other physical structural determinations.

The three penetrene classes for enveloped virus membrane glycoproteins were established based on structural similarities in the post-fusion configurations. Therefore, it is likely that there is a common post-fusion (low pH) configuration of class III penetrenes, and that GP64 superfamily members have a post-fusion structure similar to VSV G. In contrast, the prefusion configurations of class I, II and II penetrenes are highly variable. The virion configuration of VSV G is homotrimer arranged in a tripod shape with the fusion domains corresponding to the legs of the tripod [18]. No structural prediction of the prefusion configurations of GP64 superfamily members is possible.

Conversion of the virion configuration of VSV G to the fusion competent form occurs upon exposure to low pH in the infected cell. Current models suggest that low pH may permit reversible bending of VSV G at "hinge" regions flanking domain I elevating the fusion loop(s) for insertion into the host membrane [18]. Additional rearrangements of VSV G involve a rotation around the hinge, unfolding of α-helix A^0 ^and formation of helix C, interactions of the stem with domains I-III, and formation of higher multimers of the trimers. The order in which these steps occur has not been established. These changes in VSV G are hypothesized to drive deformation of the viral and target membranes. Complete cell membrane:virion membrane fusion follows, allowing entry of the ribonucleoprotein containing the viral genomic RNA. It is likely that GP64 superfamily members follow a mechanism of fusion similar to rhabdovirus G. In the case of HSV-1 gB there may be differences in the rearrangements due to size and cysteine bonding patterns of this class III penetrene [19,20]. Rearrangements involving a hinge region also occur in class II penetrenes during entry [11,12,44]. A mechanism involving rearrangement of functional domains has also been proposed for class I penetrenes [44] as well as the penetrenes of non-enveloped viruses [45]. In the case of influenza A virus HA2, the prototypic class I penetrene, the rearrangement results in formation of a trimer of the N-helices stabilized by an internal leucine zipper [42]. The leash sequence interacts with the external groove of the N-helix trimer. For other class I penetrenes the rearrange brings together the N- and C-helices into a six-helix bundle [46]. The F protein of isaviruses appears to utilize a leash-in-the groove mechanism of membrane fusion.

*Orthomyxoviridae*, *Retroviridae*, *Paramyxoviridae*, *Filoviridae*, *Arenaviridae*, and *Coronaviridae *and *Baculoviridae *have members that encode class I penetrenes [1-7,36]. Syncytin, encoded by a human endogenous retrovirus (HERV-W), is also a class I penetrene with has a critical role in membrane fusion events involved in placental morphogenesis. Syncytin may also play a pathogenic role in cancer and autoimmunity [47]. *Flaviviridae*, *Togaviridae*, and *Bunyaviridae *family members are known or appear to have members that encode class II penetrenes [10,13-15]. If the current analyses are correct, GP64 superfamily members join rhabdovirus G and herpeviruses gB as class III penetrenes. While convergence to common structures is possible, penetrenes of enveloped viruses may have evolved from a limited number of common progenitors. Support for this hypothesis comes from the remarkable similarities in the post-fusion structures of the penetrenes in each class, even though the proteins differ dramatically in aa sequence. While, it is likely that other classes of penetrenes exist for enveloped viruses, there may be a limited number of effective structures for virus-mediated membrane fusion.

**Figure 5:**
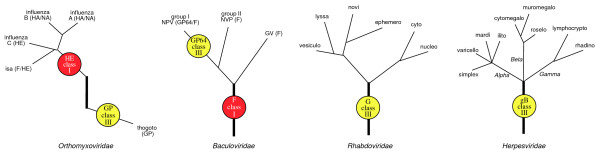
**Acquisition of class I and/or III penetrenes by members of the *Orthomyxoviridae*, *Baculoviridae*, *Rhabdoviridae *and *Herpesviridae*.** Thick lines indicate primordial lineages and thin lines are lineages leading to contemporary viruses. The orthomyxovirus tree is unrooted, while the baculovirus, rhabdovirus and herpesvirus trees are rooted. Adapted and updated from Pearson and Rohrmann [31].

Similar penetrenes are not present in all contemporary members of the *Orthomyxoviridae *[31]. Reassortment of segmented viruses is a well-establish phenomenon, and it is possible that orthomyoviruses diverged via the acquisition of segments encoding distinct penetrenes (Fig 5). The HA proteins of type A and B influenza viruses lack several structural domains present in influenza C virus HE, although the carboxyl terminal proteins (HA2 and HE2) derived from both HA and HE are class I penetrenes [48]. Members of the fifth genus in the *Orthomyoviridae*, *Isavirus*, represented by ISAV, encode two glycoproteins, HE with hemagglutinin and esterase activities and F, a class I penetrene [41]. The progenitor of the penetrenes of members of the three influenza virus genuses (A, B, and C) and ISAV may have had a segment encoding an HE-like class I penetrene that diverged to the HA in influenza A and B viruses, HE in influenza C viruses and F in isaviruses. Alternatively, HA or F could have evolved from HE or another penetrene by loss or acquisition of the esterase sequences. ISAV HE shares limited sequence similarities with the more closely related HE of influenza C viruses, coronaviruses and toroviruses [49]. Thogotoviruses appear to have acquired a distinct penetrene possibly from a common progenitor with the GP64 superfamily. It is not possible to root the tree of *Orthomyxoviridae *with regards to acquisition of penetrenes. The distinct penetrenes, class I or III, appear to have been acquired independently by different orthomyxovirus genuses, but it is unclear which, if any, was present prior to divergence of this family.

As previously discussed [31], GP64 penetrenes seem to have been acquired after divergence of the two main groups of *Baculoviridae*. Therefore, it is possible to root this tree with regards to penetrenes (Fig. 5). In this scenario, the baculovirus progenitor acquired F, a class I penetrene. One particular lineage then also acquired GP64, which we suggest are class III penetrenes, after the split into the two distinct groups of NVP and GV. Baculoviruses have large DNA genomes, and mechanisms of genetic exchange are distinct for those of RNA viruses. In contrast, the G gene appears to have been present in the common ancestor of all members of the *Rhabdoviridae*. The similarities detected between GP64 superfamily members and rhabdovirus G are consistent with divergent evolution from a common progenitor, but sequence similarities are insufficient to establish a phylogenic relationship. It is unlikely that there are any recent common ancesters of rhabdoviruses and baculoviruses, and that the class III penetrenes of these viruses were acquired by independent genetic events. The gB of herpesviruses of birds, mammals and reptiles have a high degree of conservation, and are likely to all represent class III penetrenes [19]. A gB-like progenitor probably was present in the common ancestor of these herpesviruses. Other viral glycoproteins (gC, gD, gH/gL) are involved in herpesvirus fusion and entry [50]. These additional entry proteins are differentially distributed among members of the *Herpesviridae*, and it is likely that they were acquired after acquisition of gB by the herpesvirus progenitor. Herpesvirus gB is nearly twice as long as VSV G or GP64 superfamily proteins. Assuming that the structure of gB is not an extreme example of convergence to a class III penetrene structure, it appears to have undergone extensive insertions of sequences from a common class III progenitor. Alternatively, the class III progenitor could have been a longer protein that deleted sequences prior to independent acquisitions by rhabdoviruses, thogotoviruses or baculoviruses.

## Competing interests

The author(s) declare that they have no competing interests.

## Authors' contributions

CEG performed sequence alignments and assisted in the preparation of figures. RFG supervised the work and wrote the manuscript. All authors read and approved the final manuscript.
